# Supplementary material for: Respiratory Syncytial Virus Infection Does Not Induce Epithelial-Mesenchymal Transition
Source: J Virol. 2023 Jun 20;97(7):e00394-23. doi: 10.1128/jvi.00394-23 (PMC10373540; doi:10.1128/jvi.00394-23)
Supplement: Supplemental file 10 — Fig. S1 to S9 and legends of Tables S1 to S9. Download jvi.00394-23-s0001.pdf, PDF file, 10.4 MB [file jvi.00394-23-s0001.pdf]

RSV infection does not induce EMT.

Sattya N. Talukdar[1], Brett McGregor[1], Jaspreet K. Osan[1*], Junguk Hur[1], and Masfique Mehedi[1#]

Department of Biomedical Sciences, University of North Dakota School of Medicine & Health Sciences, Grand Forks, North Dakota, United States of America.

Supplementary information:

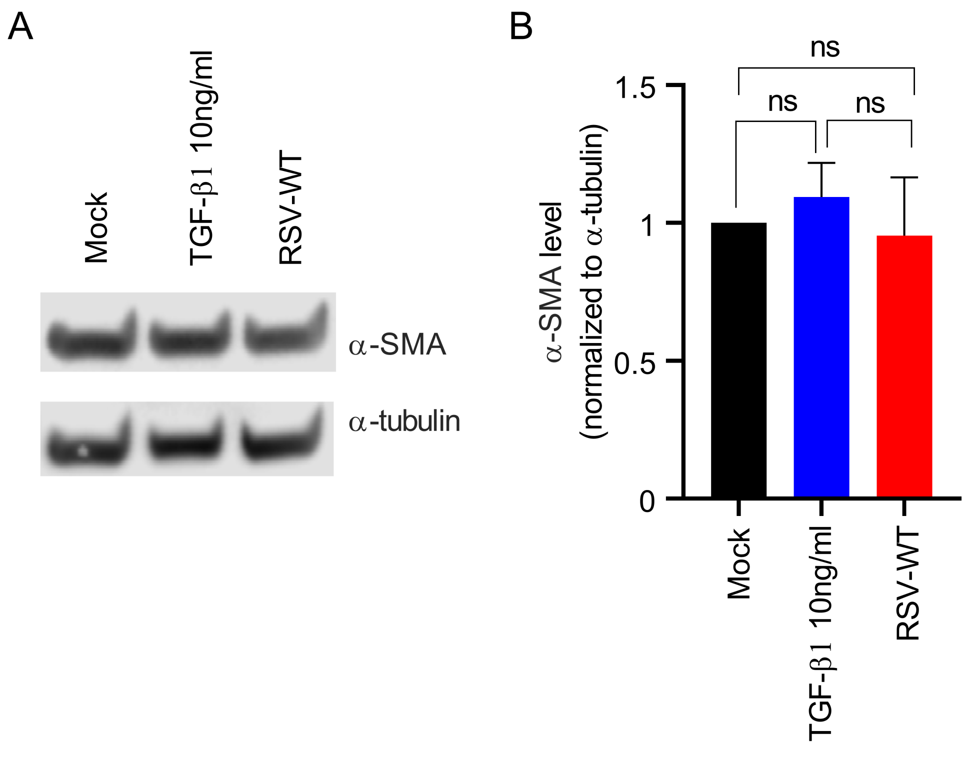

Fig. S1. RSV infection does not increase α-SMA expression in A549 cells. A549 cells were mock-infected or infected with RSV-WT (MOI = 0.1) for 2 days. Separately, A549 cells were treated with TGFβ1 (10 ng/mL) as a control. **(A)** The cells were collected and lysed. Ten micrograms of total protein was run on a reducing 4% bis-tris gel. α-SMA was detected by Western

blotting using an α-SMA-specific primary antibody and the corresponding secondary antibody. Similarly, α-tubulin was also detected as a loading control. **(B)** Relative quantification of total α-SMA (normalized to α-tubulin) in A549 cells. The data were obtained by combining results from three independent experiments, and the error bars represent SEM. One-way ANOVA was performed to determine statistical significance.

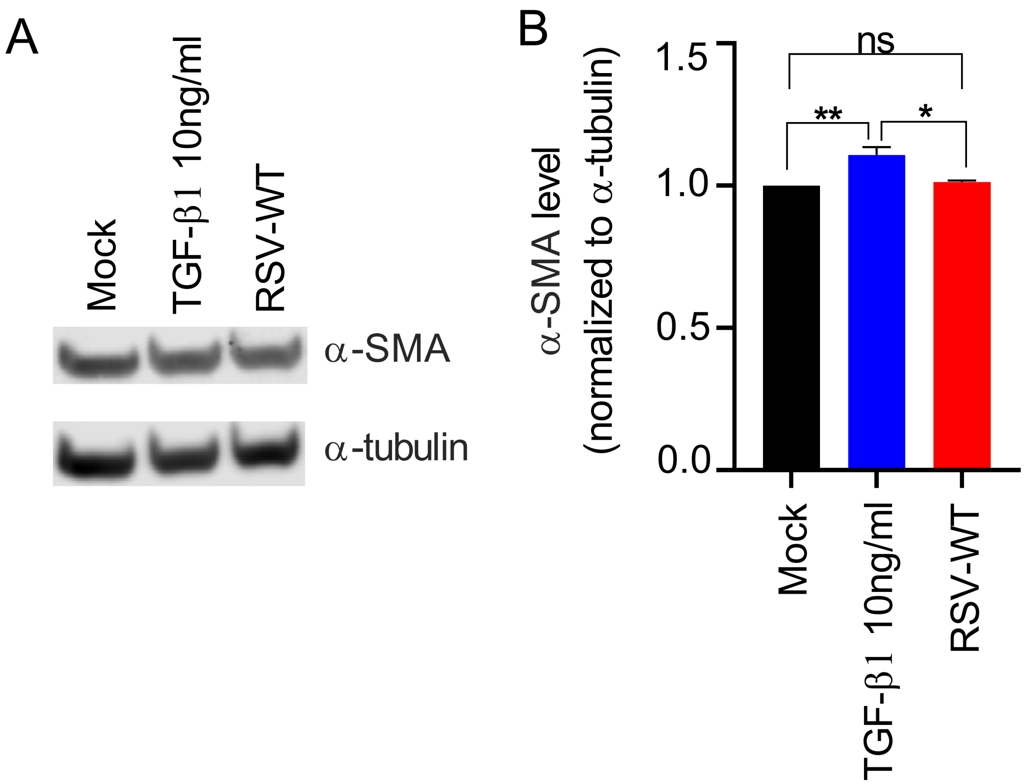

Fig. S2. RSV infection does not increase α-SMA expression in primary epithelial cells. NHBE cells were mock-infected or infected with RSV-WT (MOI = 0.1) for 2 days. Separately, NHBE cells were treated with TGFβ1 (10 ng/mL) as a control. (A) The cells were collected and lysed. Ten micrograms of total protein was run on a reducing 4% bis-tris gel. α-SMA was detected by Western blotting using an α-SMA-specific primary antibody and the corresponding secondary

antibody. Similarly, α-tubulin was also detected as a loading control. (B) Relative quantification of total α-SMA (normalized to α-tubulin) in NHBE cells. Data were obtained by combining results from three independent experiments, and the error bars represent SEM. One-way ANOVA was performed to determine statistical significance.

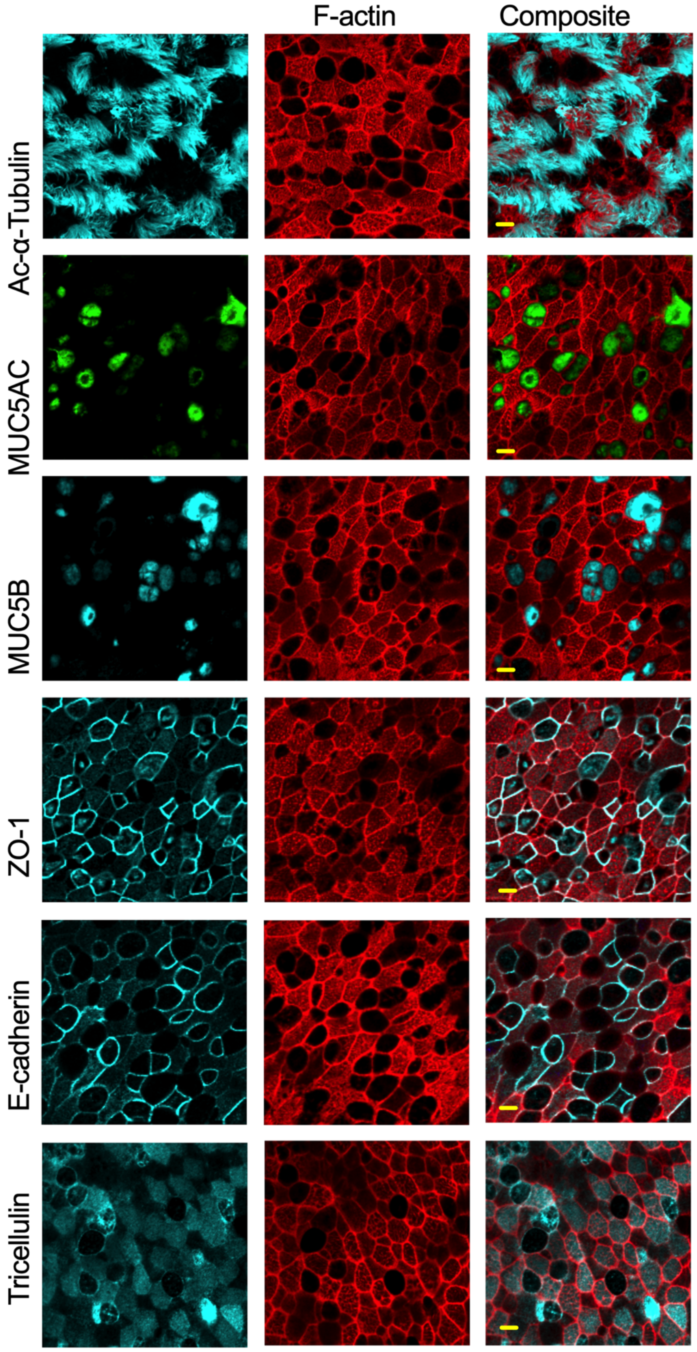

Fig. S3. Morphology and junctional characteristics of the differentiated airway epithelium. We confirmed multicellular epithelium by detecting two important cell types (ciliated and goblet cells) that were identified by cell-specific surface markers: for ciliated cells, we used acetyl-α-tubulin (cyan). For goblet cells, we used both MUC5AC (cyan) and MUC5B (cyan). We confirmed tissue-like airway epithelium by detecting adherens, tight, and tricellular junctions by E-cadherin (cyan), ZO-1 (cyan), and MALVELD2 (cyan) staining, respectively. The cell cytoskeleton was visualized by rhodamine phalloidin (red) staining. Images were captured with a 60× objective. The scale bar is 5 µm.

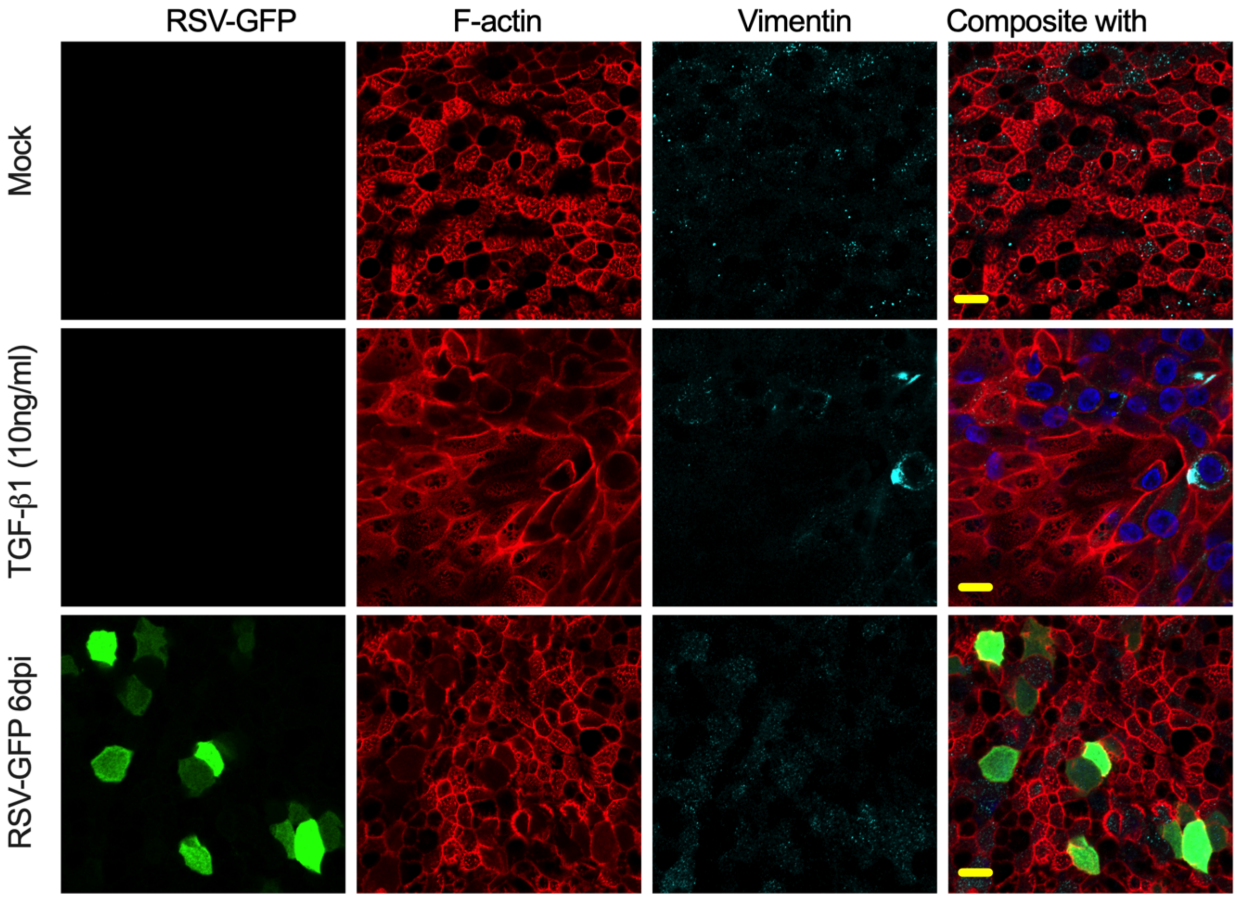

Fig. S4. Vimentin expression determination in mock-infected or mock-treated, TGFβ1-treated or RSV-GFP-infected bronchial epithelium. The cells were fixed, permeabilized, and

immunostained for vimentin (cyan) by incubating with rabbit monoclonal antibody followed by the secondary antibody anti-rabbit Alexa Fluor 647. The infected cells were identified based on the GFP signal. F-actin and nuclei were visualized by rhodamine phalloidin (red) and DAPI (blue) staining, respectively. Images were captured with a 60× objective. The scale bar is 10 µm.

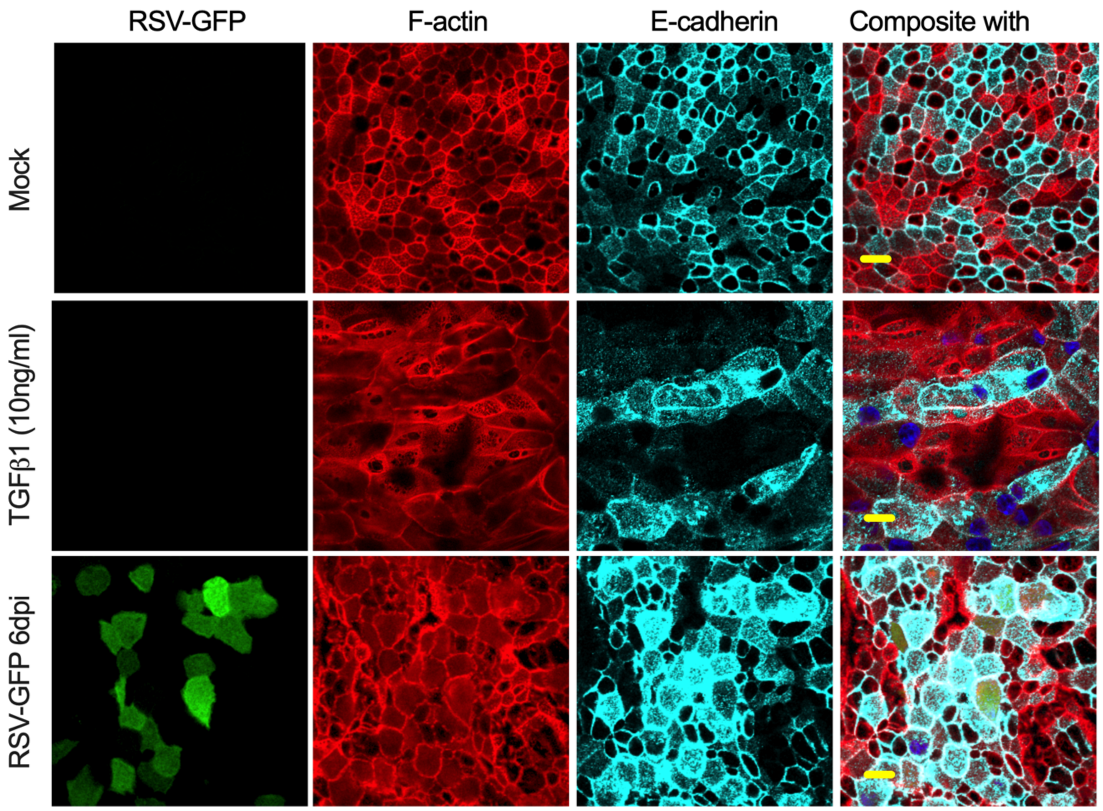

Fig. S5. E-cadherin expression determination in mock-infected or mock-treated, TGFβ1-treated or RSV-GFP-infected bronchial epithelium. The cells were fixed, permeabilized, and immunostained for E-cadherin (cyan) by incubating with rabbit monoclonal antibody followed by secondary antibody anti-rabbit Alexa Fluor 647. The infected cells were identified based on the GFP signal. F-actin and nuclei were visualized by rhodamine phalloidin (red) and DAPI (blue) staining, respectively. Images were captured with a 60X objective. The scale bar is 10 µm.

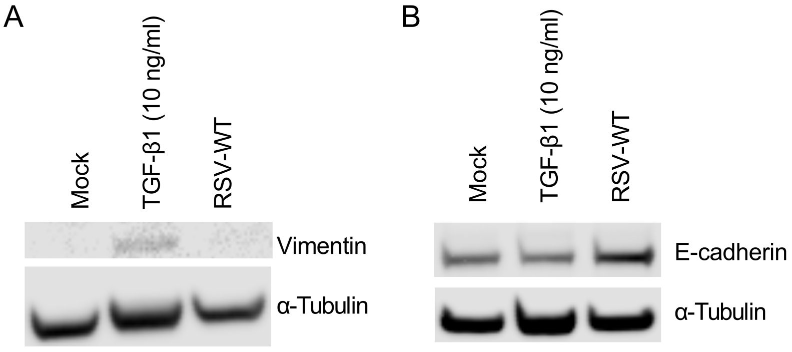

Fig. S6. RSV-WT infection neither increased vimentin nor decreased E-cadherin expression in the respiratory epithelium. The differentiated bronchial epithelium was mock-infected or infected with RSV-WT (MOI = 4) for 6 days. Separately, the bronchial epithelium was treated with TGFβ1 (10 ng/mL) as a control. **(A)** The cells were collected and lysed. Ten micrograms of total protein was run on a reducing 4% bis-tris gel. Vimentin was detected by Western blotting using a vimentin-specific primary antibody and corresponding secondary antibody. Similarly, α-tubulin was also detected as a loading control. **(B)** E-cadherin was similarly detected. The data represent one independent experiment.

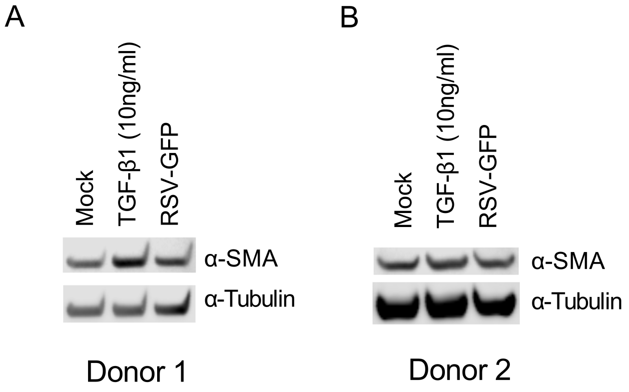

Fig. S7. RSV infection does not increase α-SMA expression in the respiratory epithelium. The differentiated bronchial airway epithelium (Donor 1, NHBE C16 and Donor 2, NHBE E16)

was mock-infected or infected with RSV-GFP (MOI = 4) for 6 days. Separately, the epithelium was treated with TGFβ1 (10 ng/mL) as a control. **(A)** The cells were collected and lysed. Ten micrograms of total protein was run on a reducing 4% bis-tris gel. α-SMA was detected by Western blotting using an α-SMA-specific primary antibody and the corresponding secondary antibody. Similarly, α-tubulin was also detected as a loading control. **(B)** Relative quantification of total α-SMA (normalized to α-tubulin) in NHBE cells. The data represent one independent experiment.

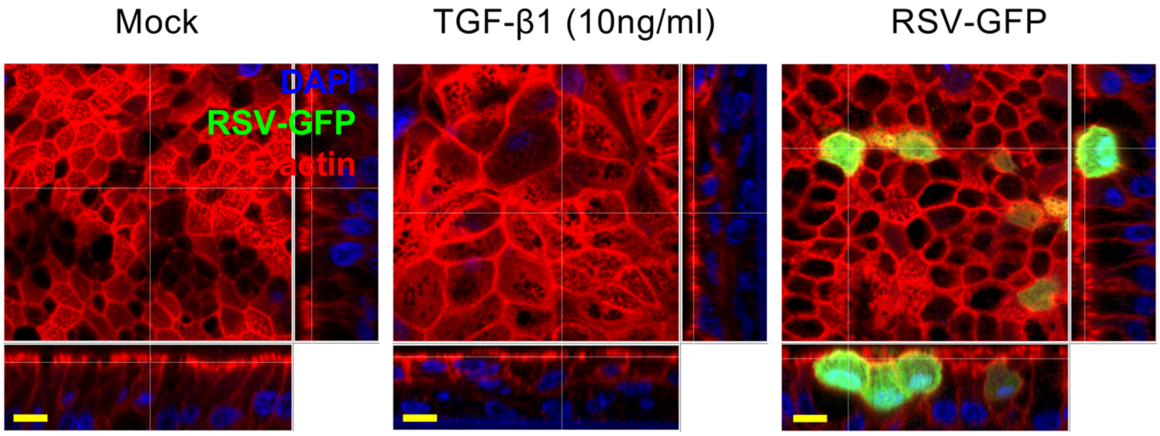

Fig. S8. Differences in epithelial morphology between RSV infection and TGF-β1 treatment. Mock-infected or mock-treated (left), TGFβ1 (10 ng/ml)-treated (middle), or RSV-GFP-infected (MOI = 4, 6 dpi) (right) samples. RSV-infected cells were detected by the GFP signal. F-actin was visualized by rhodamine phalloidin (red). Images were captured with a 60× objective and then magnified (2.5×). The scale bar is 10 μm.

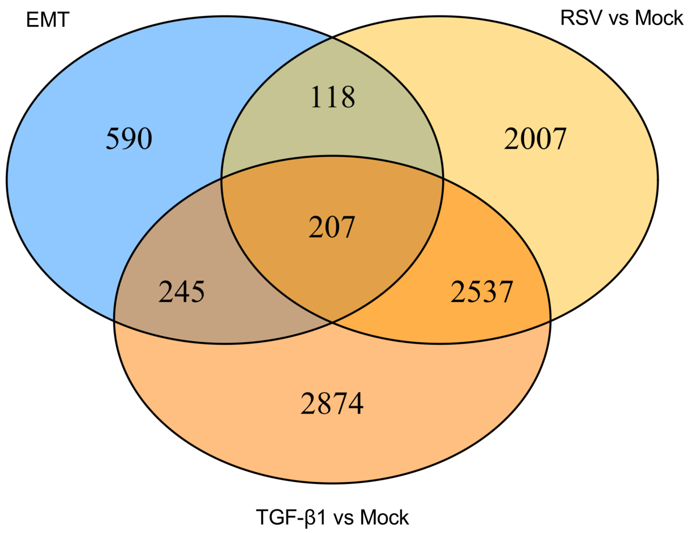

Fig. S9. Common EMT genes regulated by either RSV infection, TGF-β1 treatment or both. A Venn diagram demonstrates that both RSV and TGF-β1 commonly modulate 207 EMT genes derived from the dbEMT database.

Table S1. Differentially expressed genes (DEGs).

Table S2. RSV-specific unique gene modulation in the ranking of DEGs.

Table S3. TGF-β1-specific unique gene modulation in the ranking of DEGs.

Table S4. Enriched GO terms in RSV-specific DEGs.

Table S5. Enriched GO terms shared by both TGF-β1-specific and RSV-specific cells.

Table S6. Enriched GO terms in TGF-β1-specific DEGs.

Table S7. Genes shared by both TGF-β1-specific and RSV-specific effects.

Table S8. Gene list of the EMT database dbEMT2.0. And, overlapping with both TGF-β1-specific and RSV-specific DEGs.

Table S9. Enriched GO terms linked to Clusters 1-3.
